# Advances of Microneedles in Biomedical Applications

**DOI:** 10.3390/molecules26195912

**Published:** 2021-09-29

**Authors:** Jie Xu, Danfeng Xu, Xuan Xuan, Huacheng He

**Affiliations:** 1 Department of Dermatology, The First Affiliated Hospital of Wenzhou Medical University, Wenzhou 325000, China; skyliker@126.com; 2 Key Laboratory of Biotechnology and Pharmaceutical Engineering, School of Pharmaceutical Sciences, Wenzhou Medical University, Wenzhou 325035, China; hehc@wzu.edu.cn; 3 Allergy Center, Department of Dermatology, Zhejiang Provincial People’s Hospital Affiliated People’s Hospital Hangzhou Medical College, Hangzhou 310014, China

**Keywords:** microneedle, classification, manufacture, biomedical application, pitfalls

## Abstract

A microneedle (MN) is a painless and minimally invasive drug delivery device initially developed in 1976. As microneedle technology evolves, microneedles with different shapes (cone and pyramid) and forms (solid, drug-coated, hollow, dissolvable and hydrogel-based microneedles) have been developed. The main objective of this review is the applications of microneedles in biomedical areas. Firstly, the classifications and manufacturing of microneedle are briefly introduced so that we can learn the advantages and fabrications of different MNs. Secondly, research of microneedles in biomedical therapy such as drug delivery systems, diagnoses of disease, as well as wound repair and cancer therapy are overviewed. Finally, the safety and the vision of the future of MNs are discussed.

## 1. Introduction

**Figure 1:**
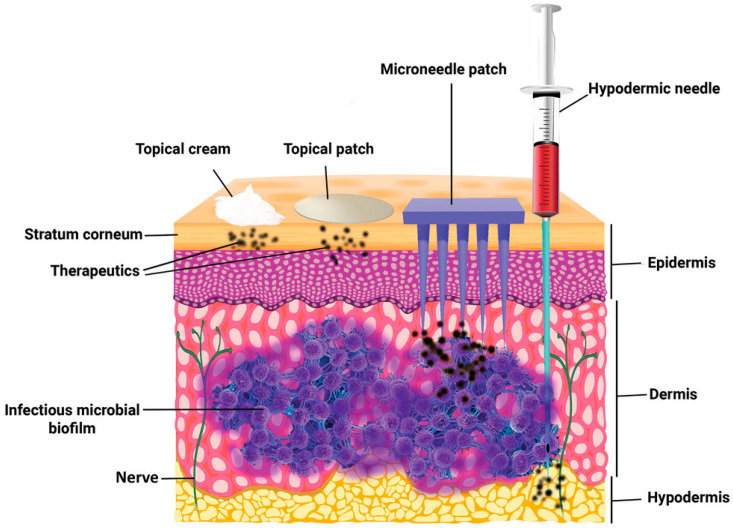
The comparison of skin penetration depths across different drug delivery systems. (Image was reproduced with permission from [2]).

Pain associated with conventional injection for drug administration causes poor adherence. Oral administration is convenient, but the efficiency of drug delivery is limited by the first-pass effect. A microneedle is a novel approach to drug delivery that overcomes these limitations. Microneedles can physically penetrate the stratum corneum and create micropores larger than macromolecular drugs, thus providing direct channels for drug diffusion. Alternatively, drugs can be loaded in hollow microneedles and directly injected into the circulatory system. Drugs can also be mixed with a soluble substance that penetrates the skin and reaches the circulatory system. Microneedles can be used for transdermal and non-transdermal drug delivery. In transdermal drug delivery, microneedles can reduce or eliminate the pain of injection. The length of microneedles ranges from 25 to 2000 μm [1] and can penetrate the skin barrier without damaging neural tissue (Figure 1) [2]. Furthermore, microneedles is a kind of continuous administration, so that it can reduce the frequency of administration, especially for insulin injection in patients with diabetes [3,4]. Non-transdermal administration is used for buccal mucosa [5] and surgery-exposed tissues such as eyeballs [6], vascular tissue [7] and the gastrointestinal tract [8]. For example, in 2011, Patel et al. injected particles into the suprachoroidal space through hollow microneedles and delivered a drug to the back of the eye [9].

**Figure 2:**
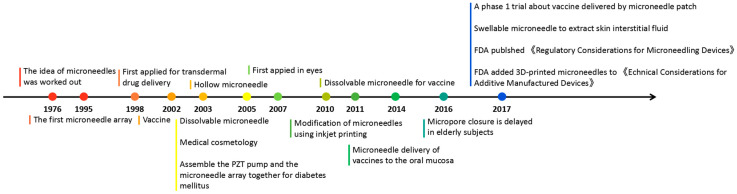
Timeline of microneedle development. The time point listed represented the important events associated with microneedles from the idea of microneedles in 1976.

**Figure 3:**
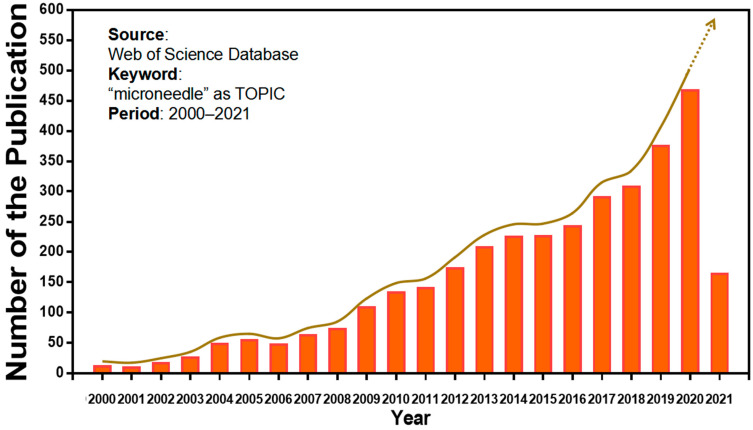
Keyword literature search of microneedles in Web of Science. The popularity of microneedles has been growing every year from 2000 until now.

Microneedles were developed in 1976 and used for transdermal drug delivery in 1998. They have been intensively studied over the past half-century (Figure 2) [10,11,12,13,14,15,16,17,18,19,20,21]. A “Web of Science” search with the topic of “microneedle” yielded 3407 articles published since 2000 (Figure 3), which indicates the rapid increase of microneedle studies. Moreover, most microneedles are applied in biomedical areas, especially for cancer therapy, skin disease therapy, insulin delivery for diabetes treatment, blood glucose level detection and vaccines [17,22,23,24,25,26,27,28,29,30]. Therefore, in this work, we mainly review the classification, manufacture and biomedical applications of microneedles. The pitfalls of the application of microneedles are also summarized. Given the advantages of microneedle administration, it is promising to optimize the fabrications of microneedles and avoid their disadvantages in future studies.

## 2. Classification of Microneedles

**Figure 4:**
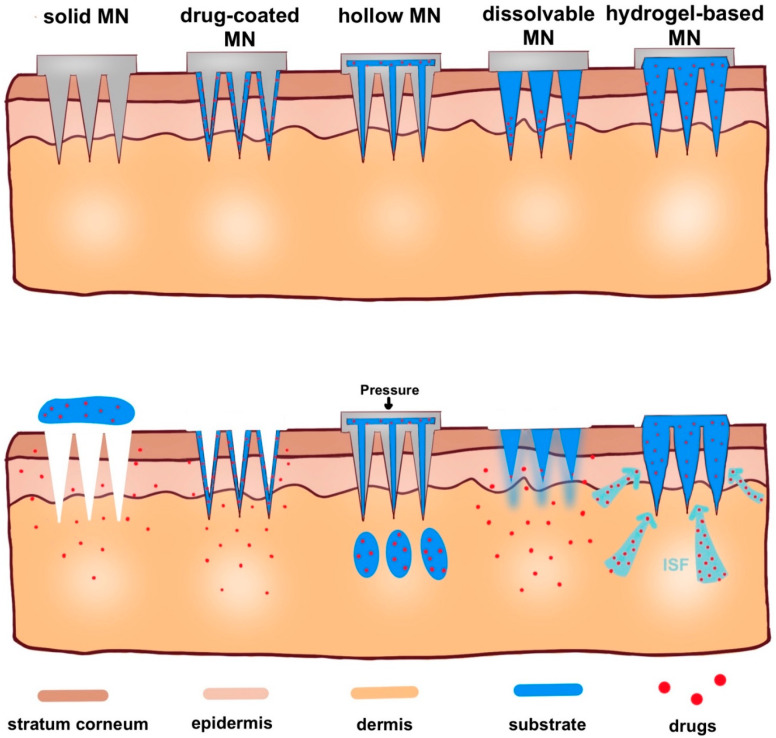
Schematic representation of five types of microneedle administration methods. Solid MNs are inserted into the skin and removed, leaving a channel through which the drug enters. Drug-coated MNs are the same as solid MN, except the drug is on the surface of the microneedles. For hollow MNs, after adding pressure, drug is released from the hollow microneedle. For dissolvable MN, when the microneedle substrate is dissolving, the drugs on the tip of the needle are released. When hydrogel-based MNs swell up from absorbing (ISF), the drug is released into the body.

Here, in this review, according to microneedle-based devices, MNs can be divided into five categories: solid, drug-coated, dissolvable, hollow and hydrogel-based microneedles (Figure 4) [31,32].

### 2.1. Solid Microneedles

Solid microneedles are generally made of metal, silicon or polymer for pretreatment of fine incisions and improvement of drug permeability. Sabri et al. found that solid microneedles promoted the ex vivo transdermal absorption of imiquimod (one of the most effective drugs for the treatment of basal cell carcinoma), and the intradermal depot persisted for up to 24 h [33]. However, the application of solid microneedles requires two steps, and they may not be convenient for patients.

### 2.2. Drug-Coated Microneedles

Drug-coated microneedles can be used for delivering active molecules such as small molecules [34], proteins [35] and vaccines [36]. At the same time, drug-coated microneedles takes advantage of remaining active for a long time while the dose of the coated drug may be low [37]. Moreover, the drug amount loaded in the coated MNs system is limited. For instance, a maximum of 1 mg of drug can be coated on MNs, while much larger amounts of drugs (up to 33 mg) can be delivered by dissolvable MNs [38].

### 2.3. Dissolvable/Biodegradable Microneedles

**Figure 5:**
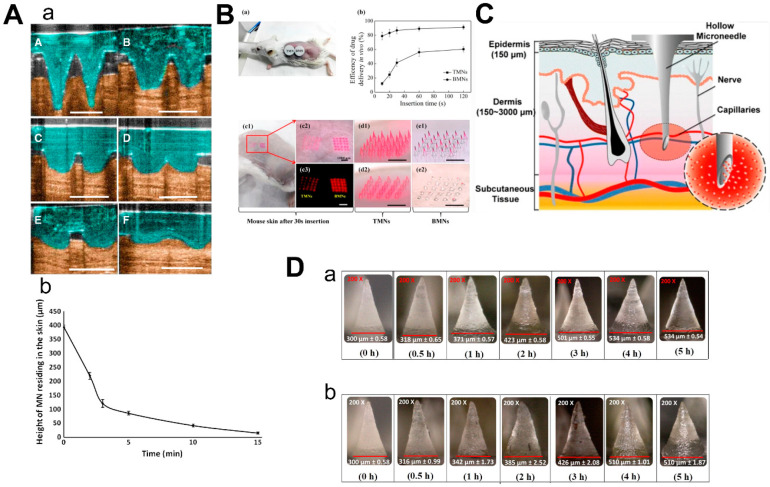
Characteristics of 3 types of microneedles. (**A**) Diagram (a) and dissolution curve (b) of dissolvable microneedles in mice. (**B**) Pharmacokinetics of TMN and BMN in mice with different inserting times (a,b) and the comparison of images before and after TMN and BMN are inserted into the skin (from 10 s to 120 s). TMN: traditional microneedle. BMN: bubble microneedle. (**C**) Schematic diagram of hollow microneedles for blood extraction. (**D**) Swelling images for hydrogel-based MNs loaded with α-arbutin in porcine skin (ex vivo) and in mice skin (in vivo) at different time points from 30 min to 5 h. (Images were reproduced with the permission from [40,41,43,44]).

Dissolvable/biodegradable microneedles dissolve completely upon insertion into the skin and have high biocompatibility—because safe polymers can be used as raw materials—and have high loading capacity [39]. When soluble microneedles were used on mouse ears, the length of microneedles within the skin rapidly decreased to 25% of the initial depth within the first 3 min and then slowly but constantly dissolved over the next 10 min (Figure 5A) [40]. This feature can be used for sustained drug delivery, but it also suggests that patients may need to wait for the microneedles to dissolve in the skin. Thus, Wang et al. designed a kind of microneedle which had bubble-structure microstructures in the body of the needle. The bubble structure promotes the concentration of the drug to the needle tip and enables the drug delivery efficiency to reach over 8% in 20 s in mice (Figure 5B) [41]. However, attention should be paid to whether the mechanical characteristics can be maintained in a humid environment [42].

### 2.4. Hollow Microneedles

Hollow microneedles can be used to inject solutions or suspensions to provide specific channels for therapies targeting specific tissues. Li et al. reported a method to optimize the manufacturing process of hollow microneedles to produce long and sharp microneedles that could reach the vessels for blood analysis (Figure 5C) [43]. Furthermore, hollow microneedles have a good command of the amount of drug and control of the time of the drug’s release, while fabrication was difficult and had risks linked to needle breakage and lumen blockage [45].

### 2.5. Hydrogel-Based Microneedles

The mechanism of hydrogel-based MN drug delivery is the same as dissolvable MN: insert into the skin, release the drugs and there is no need to discard the needle [31]. Due to the hydrophilic nature of the hydrogel, the hydrogel-based microneedles will absorb the interstitial fluid and swell when they are inserted into the skin [46]. Materials with biocompatibility and good swelling properties should be selected to make hydrogel-based microneedles [47]. Aung et al. investigated the swelling of the poly(vinyl-alcohol) (PVA) combined polyacrylic acid-co-maleic acid (PAMA) hydrogel-based microneedles at different times after they were inserted into the skin of mice and pigs, respectively (Figure 5D). Although the microneedles were slightly deformed, they still kept mechanical strength. Compared to dissolvable MN, hydrogel-based microneedles could load a larger amount of drugs [44].

## 3. The Manufacture of Microneedles

Microneedles are made of silicon, metals and polymers. Silicon is easily shaped but prone to fracture, and it requires a clean space and a high cost of manufacturing [48]. Metals are less expensive but produce wastes and biohazardous materials. Polymers are highly viscous, not prone to fracture, and are mostly biocompatible and suitable for low-cost mass production [49], which has been extensively used to fabricate microneedles for biomedical applications. Several biocompatible materials have been developed for microneedle fabrication, such as carboxymethyl cellulose (CMC) [50], PVA, polyvinylpyrrolidone (PVP), poly (lactic-co-glycolic acid) (PLGA) [51], hyaluronic acid (HA), methacrylated hyaluronic acid (MeHA) [52] and so on. Mao et al. loaded poorly water-soluble rapamycin into PVP MN, as PVP can help rapamycin dissolve quickly in the body. Furthermore, when PVP MN and human umbilical vein endothelial cells were incubated together for 48 h, the survival rate of the cells was still over 90%, suggesting excellent biocompatibility [53]. Yao and co-workers observed the degradation of MeHA-MN in a simulated in vivo environment (PBS buffer solution with 2.6 U mL^−1^ hyaluronidases, 150 rpm, 37 °C). When MeHA concentration is 3%, MeHA-MN could obtain good mechanical strength and gradually degrade within 8 days. Besides, they found that the MeHA-MN group had a significantly higher cell activity (NIH-3T3 cells) than the control group [54].

Thus, here we mainly introduce the polymer-based manufacturing methods for microneedle preparation. There are two main categories of polymer manufacturing methods, drawing lithography and micromolding, which have similar procedures. First, the material is molded with or without a mold and then hardened by external stimuli. Drawing lithography is a simpler method to fabricate microneedles without molds. The polymer is firstly put on a temperature-controlled lower substrate. The upper substrate with a series of cylinders is in contact with the polymer-added lower substrate. Then, the upper one is lifted to make the polymer stretch, which forms microneedle structures that can be solidified by heat [55], electric field [56], magnetic field [57], or centrifugal force [58]. When a mold is used, the shape of microneedles can be solidified by crosslinking with heat or ultraviolet (UV) light [59]. Last but not least, three-dimensional (3D) printing is also another way to manufacture MNs. Firstly, computer-aided design (CAD) software was used for virtual design, and then the virtual model was input into a 3D printer. At last, 3D printing can provide high-throughput manufacturing of physical objects with high precision and reproducibility [60].

### 3.1. Drawing Lithography

**Figure 6:**
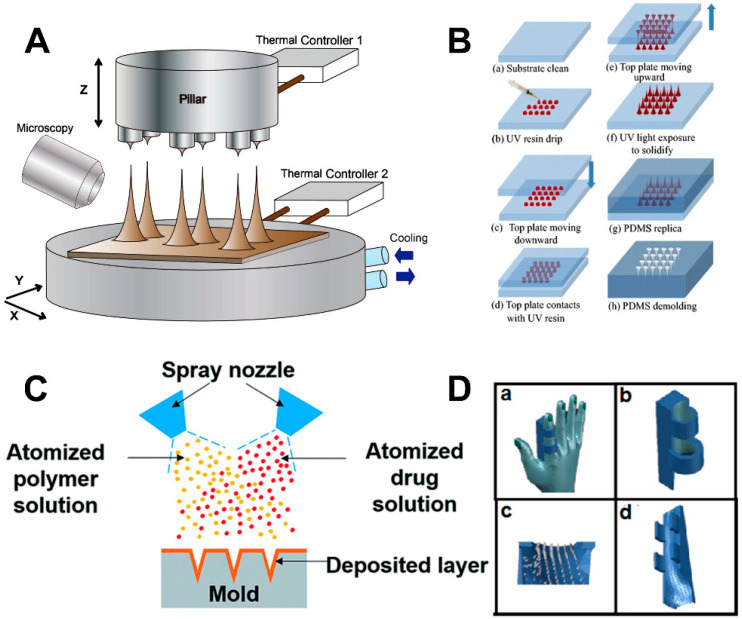
Illustration of methods for making microneedles under different conditions. (**A**) The shape of the microneedle is fixed by heating–cooling thermal controller. (**B**) Fabrication process of the microneedle mold by UV. (**C**) Scheme of dual-nozzle spray deposition process. (**D**) Scheme of producing a personalized microneedle by 3D printing. (Images were reproduced with permission from [55,59,62,63]).

Drawing lithography takes the viscous polymer in the glass transition process as a crucial point to achieve the manufacturing performance of a 3D microstructure [61]. In the thermal drawing of microneedles, the biodegradable thermoplastic poly(lactic-co-glycolic acid) (PLGA) is vertically stretched by a metal pillar while the speed is controlled. The top is broken by fast drawing to form a microneedle structure, and the shape of the microneedle can be adjusted by changing the temperature and fracture speed after cooling. In this process, it is necessary to ensure that the properties of the material remain unchanged after heat treatment (Figure 6A) [55]. For example, a magnetorheological drawing lithography method is proposed by Chen et al. to form a flexible microneedle array. It only needs one step, which is formed from the compressed droplets of curable magnetorheological fluid on a flexible substrate. Then, the MN was solidified in an oven for 1 h with a temperature of 90 °C, and the penetration of calcium protein through rabbit skin increased after the application of this microneedle [57]. In addition, drawing lithography is suitable for mass production due to its one-step manufacturing process. 

### 3.2. Micromolding

In micromolding, it is necessary to have a mold of certain specifications, which is generally made of resin. Lin et al. solidified resin by using 30–40 s exposure to UV light with different power outputs. Then, the solidified structures were replicated with polydimethylsiloxane (PDMS) to make a microneedle array mold, which can be filled with water-soluble or biodegradable materials (Figure 6B) [59]. Yu et al. made the microneedles using two steps, casting and coating. They obtained amifostine-loaded MN patches filled in the mold and then put them under vacuum at room temperature and dryed them for 2 min. Finally, the surface of MN was immersed in a solution containing UV light-irradiated 1% trimethylbenzoyl phosphine oxide [64]. However, solutions with high viscosity are easy to produce bubbles after filling the mold, while lowly viscous solutions may lead to microneedles so thin that their physical properties are reduced. Hwa et al. had determined the appropriate viscosity by observing the flow condition of different concentrations of CMC in the test tubes. After choosing 3% CMC, the solutions were centrifuged, poured into molds and placed in a vacuum (65 °C, 24 h) [50]. After filling in the mold, vacuum or centrifugation was applied to avoid the constraints of surface tension and viscosity of the solution, which require more steps in the micromolding processes. Marie et al. used an atomized spray process to minimize the effects of the liquid surface tension and viscosity when filling molds [65]. Kim et al. fabricate MN patches by a dual-nozzle spray deposition process, which improves the stability of drugs by eliminating the emulsification as well as reducing adverse interaction with solvents (Figure 6C) [62].

### 3.3. Three Dimensional (3D) Printing Technology

Three-dimensional printing is an emerging manufacturing technique based on 3D model data in computers; it uses a method of layer-by-layer accumulation of materials. This printing technique has the advantages of high accuracy, high precision, high flexibility, fewer manufacturing steps and less waste. Currently, the 3D printing techniques used to manufacture microneedles for transdermal drug delivery mainly include 3D projection inkjet (3DPI), fused deposition molding (FDM) [66], photopolymerization-based approaches (stereolithography (SLA) [67]; two/multi-photon polymerization (2PP/MPP); digital light processing (DLP) [68]) and laser-assisted bioprinting (LAB). Although MPP has the highest resolution, it was limited by speed and materials. Meanwhile, SLA has a higher resolution but with more impact by oxygen inhibition than DLP [69]. Three-dimensional printing has been integrated into microneedle fabrication via photopolymerization. According to Shin et al., DLP-based 3D printing was adopted to construct molecules in an aqueous solution with lower concentration by photo-crosslinking, combining silk fibroin with riboflavin to form a flexible MN [70]. Given the low drug load of microneedles and the high flexibility of 3D printing, Seng et al. fabricated a dual-function microneedle array on personalized curved surfaces for drug delivery and splint to treat the affected finger by DLP. The microneedles can withstand twice the average thumb force without breaking, and the amount of drug that penetrated the skin significantly increased (Figure 6D) [63]. Inkjet printing is a non-contact process for the on-demand delivery of biological materials containing small amounts of proteins and nucleic acids [71], which can be used to manufacture microneedles with improved coat uniformity, stability and reproducibility. For instance, Pere et al. made MNs with biocompatible resin by using a stereolithography printer, and then printed the insulin solution to the surface of MNs through the inkjet printer, which is similar to the atomized spray process mentioned above [38]. 

## 4. Applications

**Figure 7:**
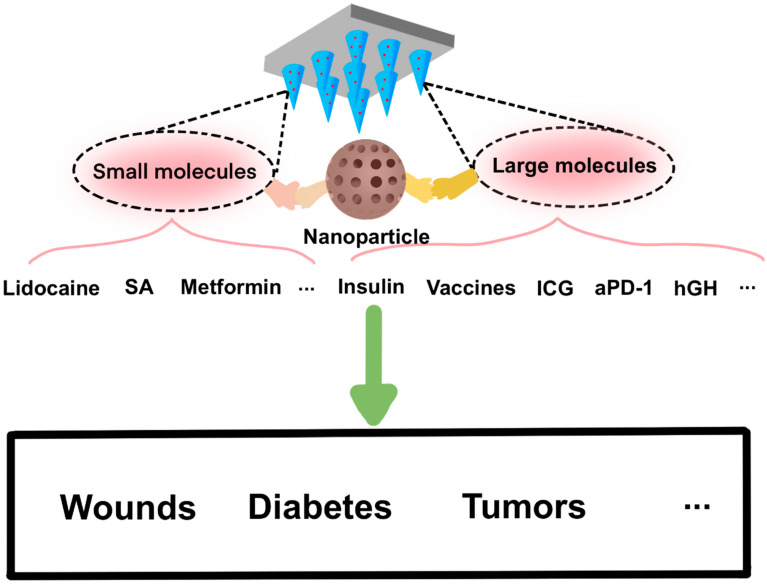
Schematic representation of MNs with small and large molecules in the application of wounds repair, diabetes and tumor therapy.

A large number of studies have demonstrated the application of microneedles since the initial report in 1976. In general, the application of microneedles in biomedicine falls into two categories: therapy and diagnosis. Some drugs can be delivered through the skin with the use of microneedles. Numerous studies have shown that microneedles can be used for transdermal delivery of metformin [72], lidocaine [73], insulin [74], vaccines [17], human growth hormone (hGH) [75] and nanoparticles (NP) [76] (Figure 7) in the treatment of wounds (acne) [77], diabetes therapy [23], tumors [78] and so on. In terms of diagnosis, microneedle-based biosensors have been intensively studied for the extraction and analysis of interstitial fluid and blood [79] and the screening of skin melanoma [80].

**Figure 8:**
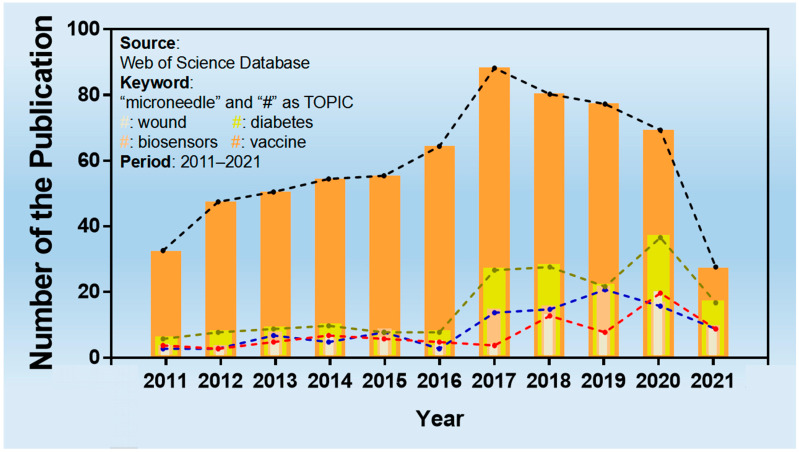
The number of publications on “microneedles” with different topics.

Besides cancer therapy, which is the same as other reviews summarized in [81], the main part of this review focuses on the application of microneedles in the areas of skin wound therapy, diabetes treatment, vaccines and sensors. A total of 978 articles published since 2011 were retrieved with the keyword “microneedle” in combination with “wound”, “diabetes”, “vaccine” or “biosensors” from Web of Science. The number of articles fluctuated but gradually increased over the years. Figure 8 summarizes the number of articles published on different topics in the last decade.

### 4.1. Wound Repair

**Figure 9:**
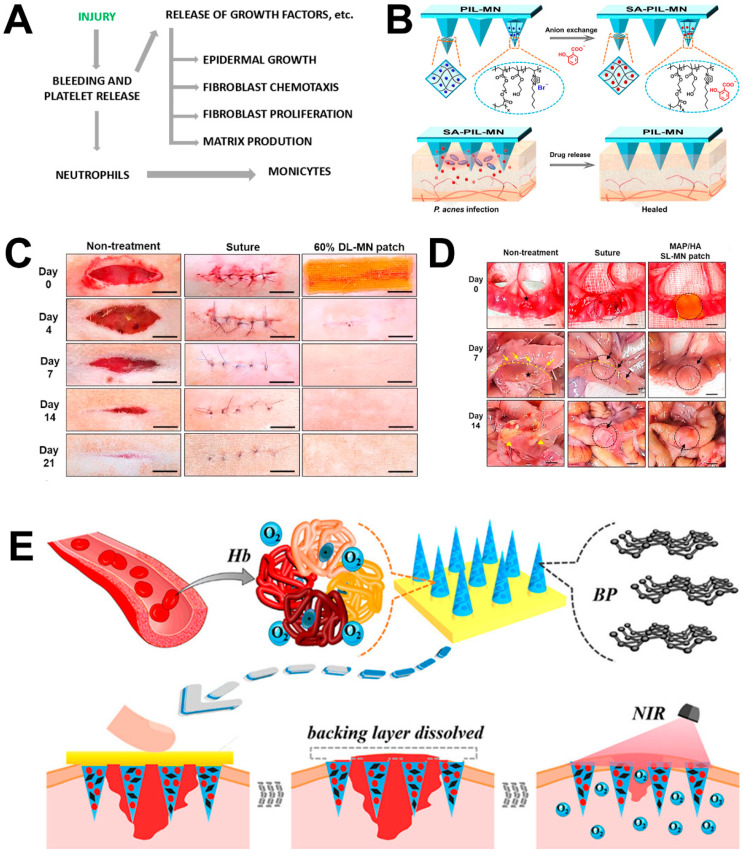
Schematic diagram of the principle and the wounds by microneedle therapy. (**A**) Inflammatory responses after microneedle was applied to the skin. (**B**) Release of SA from SA-IL-MNs. (**C**,**D**) Macroscopic images of skin or ileum wounds in DL-MN (double-layer-MN) treated rats. (**E**) Schematic diagram of the BP-loaded, oxygen-carrying responsive MNs in wound healing. (Images were reproduced with permission from [15,77,86,87]).

Acne is a common dermatological condition with excessive collagenase as a response to local skin inflammation, and the wound-healing process leads to an overall loss of collagen from the underlying lesions, resulting in atrophic (depressed) scars [82]. In addition to laser treatment and subcutaneous incision, disposable microneedle tips with adjustable depth and speed of incision can be used to cause uniform bleeding points on the skin and create multiple micro-bruises in the dermis that can trigger complex cascades of growth factors that ultimately lead to collagen production [15,83]. Collagen is converted to type I collagen via tissue remodeling and vascular maturation, which results in skin tightening and skin repair (Figure 9A). Camirand and Doucet first described the advantages of microneedles in the treatment of acne scars, and they used a tattooing machine to remove atrophic scars [84]. However, to avoid unnecessary irritation of the wound, microneedles should not be used on damaged skin when not aimed for treatment [85]. Zhang et al. developed a poly(ionic liquid)-MN (PIL-MN) patch prepared by UV light in the PDMS mold. It contained salicylic acid (SA) as the active ingredient, which inhibited *Propionibacterium acnes*., Gram-negative *E. coli* and Gram-positive *Staphylococcus aureus*. The results showed that mice treated with SA-PIL-MN had low expression of inflammatory factors; although large doses of SA alone inhibited the inflammatory response triggered by acne, the effect on acne inhibition was inferior to that with SA-PIL-MN, probably due to the good distribution and effective transdermal release of microneedles (Figure 9B) [77]. Jeon et al. developed a DL-MN patch consisting of an expandable mussel-adhesion-protein (MAP)-based shell and non-swellable silk fibronectin (SF)-based core which was also fabricated in the PDMS mold and dried for 12 h in vacuum at −85 kPa. The patch rapidly adhered, closed wounds under wet and/or dynamic conditions and showed the desirable ability in superficial and internal wound repair (Figure 9C,D) [86]. Zhang et al. loaded black phosphorus quantum dots (BP QDs) and oxygen-carrying hemoglobin (Hb) on separable microneedle tips to achieve near-infrared-controlled oxygen delivery so that oxygen was transferred directly into the deep layer of the skin. These microneedles ensured the ideal healing of full-thickness cutaneous wounds in a diabetic rat model (Figure 9E) [87]. For a porcine dorsal skin model, Park et al. designed a strategy to treat keloid by loading 5-fluorouracil (5-FU) onto carboxymethyl chitosan (CMC) nanoparticles into a solid MN. The results suggested that the side effects of 5-FU can be reduced by local administration of small molecules loaded with nanoparticles using MNs [88].

### 4.2. Diabetes Therapy

**Figure 10:**
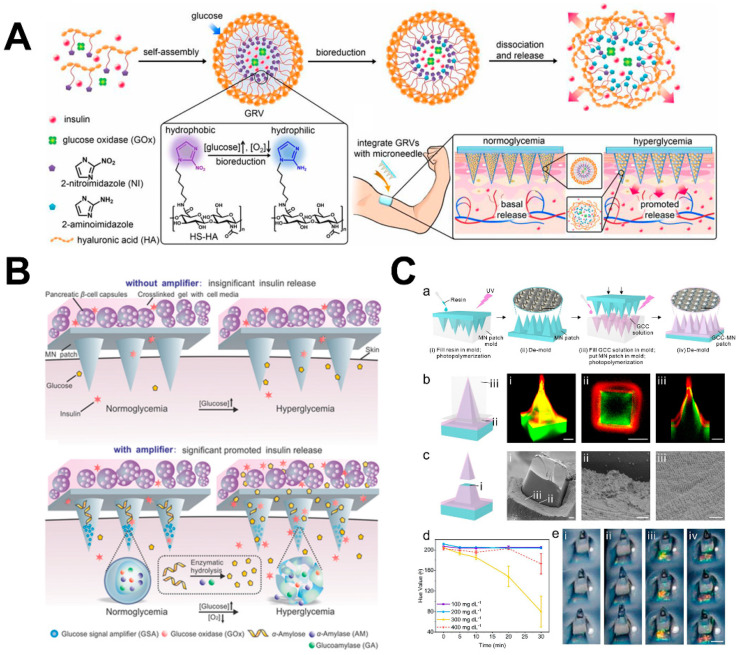
Illustration of microneedles for the diagnosis and therapy of diabetes. (**A**) Illustration of an insulin-releasing microneedle to detect glucose level. (**B**) Schematic diagram of a microneedle that can be used as a glucose signal amplifier (GSA). (**C**) Schematic diagram of painless patch for detecting blood sugar by colorimetry. (Images were reproduced with permission from [23,90,91]).

Diabetes is a chronic disease, and diabetes-associated complications affect more than 425 million people all over the world [22]. Current treatment of diabetes depends on multiple daily injections of exogenous insulin to continuously regulate blood glucose levels. However, frequent insulin injections can lead to long-term complications and poor compliance, and insulin overdose can lead to severe shock or even death [89]. Therefore, there is a pressing need for a painless, noninvasive and self-administration method that can be used repeatedly. More importantly, the dose should be adjusted according to the actual need. Moreover, as a transdermal drug delivery system, microneedles can avoid gastrointestinal irritation and first-pass effects by oral delivery. Additionally, the gastrointestinal tract is an important active site of metformin hydrochloride, while metformin can cause gastrointestinal problems, including stomach pain, vomiting and other side effects. Therefore, Migdadi et al. designed hydrogel-based microneedles to allow sustained delivery of metformin through the transdermal drug delivery system [72]. Yu et al. reported a novel glucose-responsive insulin delivery device (Figure 10A). The device has a microneedle-array patch (“smart insulin patch”) which is not only painless but also contains GRVs loaded with insulin and GOx. This device uses local hypoxia caused by enzymatic reaction as a trigger for the rapid release of insulin in response to hyperglycemia. During percutaneous administration, when exposed to high tissue fluid glucose in the blood vessel and lymphatic capillary network, the GRVs loaded in the microneedles will decompose. Continuous administration with a microneedle patch could accurately control blood glucose within the normal range for prolonged periods of time [90]. Ye et al. used a microneedle patch to regulate insulin secretion by pancreatic β-cells through glucose-responsive adjustment of blood glucose levels without implantation. Since the patch does not have an effective response to hyperglycemia, in order to stimulate an effective cell response, microneedle-based GSAs were synthesized (Figure 10B). Additionally, MN was prepared by UV crosslinking (30 s, wavelength of 365 nm), and GSA was deposited on the tip by centrifugation so that the β-cell capsules were allowed to secrete insulin through the MN [23]. Zeng et al. created a minimally invasive, painless patch for colorimetric glucose monitoring with the naked eye, namely GCC MNs, which are cured by ultraviolet light. The structure of GCC changed with the increase in glucose concentration, the reflection peak red-shifted and the color of GCC shifted from violet (100 mg dL^−1^ glucose) to blue (200 mg dL^−1^ glucose) and then to green (400 mg dL^−1^ glucose) (Figure 10C) [91].

Due to the limits of bioavailability and dose, solid MNs, dissolvable MNs and drug-coated MNs are not high-potency ways for clinical applications to deliver insulin. In 2009, an MN was first used to deliver insulin to human subjects. Jyoti et al. proved that hollow microneedles could availably deliver burst insulin to patients who had type I diabetes in a minimally invasive way [92]. Lee et al. fabricated a MN by reverse drawing lithography. Additionally, they assembled this MN with a 1 mL disposable syringe. They found that after the application of an MN and pen needle injection on 15 patients with type II diabetes and 25 healthy adults, the MN could manage blood glucose more steadily, similar a the traditional hypodermic needle [93].

### 4.3. Cancer Therapy

**Figure 11:**
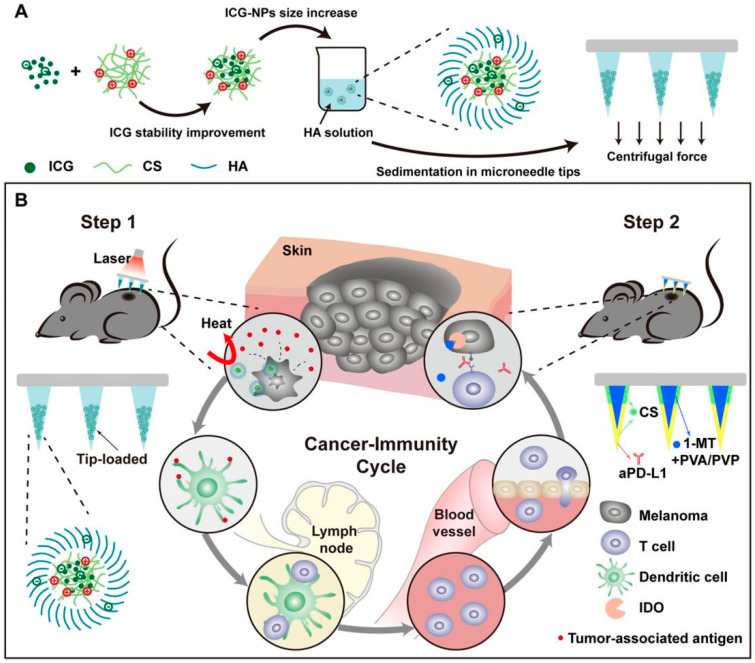
Schematic diagram of indocyanine green-nanoparticle microneedles (ICG-NP MN): preparation and treatment of melanoma cancer. (Images were reproduced with permission from [76]).

Microneedles with nanoparticles are very promising for the treatment of superficial tumors. After reporting MNs loaded with pH-responsive tumor-targeted lipid-coated cisplatin nanoparticles, Su and his colleagues further investigated a novel MN to achieve synergistic immuno-chemotherapy with antibodies anti-programmed cell death protein 1/cisplatin-diammineplatinum@NPs (aPD-1/CDDP@NPs). Notably, MNs increased the response rates in squamous-cell carcinoma mice models in which there was no response with a single aPD-1 treatment. Thus, the MNs showed promise for the treatment of cancers [94]. Similarly, Yang et al. reported a strategic topical and targeted photothermal therapy (PTT) mediated by a microneedle system (Figure 11). The indocyanine green was entrapped in chitosan nanoparticles and deposited on the tip of the needle. This indocyanine green-NP (ICG-NP) MN therapy combined with Near Infrared (NIR) irradiation could effectively inhibit melanoma growth early on by destroying most tumor cells. Furthermore, after being combining with antiprogrammed death-ligand 1/indoleamine 2,3-dioxygenase inhibitor indoximod chitosan microneedles (aPD-L1/1-MT CSMNs), tumor growth was completely inhibited within 10 days [76].

### 4.4. Vaccines

**Figure 12:**
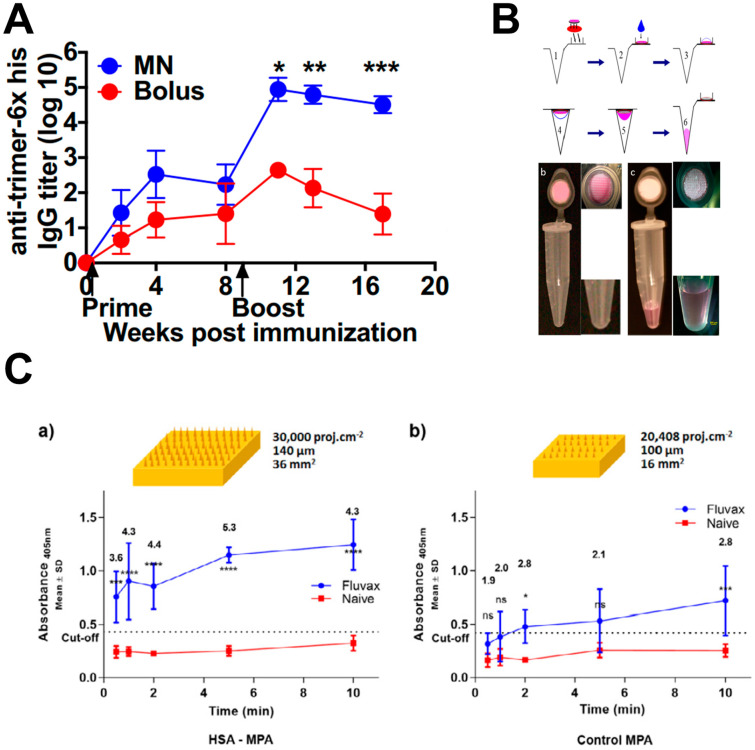
Analytical diagrams and schematics of microneedles used for vaccines and biosensors. (**A**) Enzyme-linked immunosorbent assay (ELISA) analyses of anti-trimer serum IgG responses over time in strong trimeric-specific humoral response maintained by MNs. (**B**) Procedures of microneedles to extract tissue fluid. (**C**) Fluvax IgG was captured from immunized mice to a certain extent by microneedles with different surface areas. In (**A**,**C**), 2-way ANOVA analysis is applied, and the significant difference is indicated by * *p* < 0.05, ** *p* < 0.01, *** *p* < 0.001, **** *p* < 0.0010, while n.s. indicates no significant difference. (Images were reproduced with permission from [98,101,102]).

Some vaccines are lyophilized, diluted and injected multiple times, which inevitably increases the cost and waste, as well as the probability of contamination during transportation and storage. The use of microneedles would not only reduce the costs but could also ensure a stronger immune response due to sustained administration (Table 1) [95]. Sullivan Sean P et al. used dissolvable microneedles for influenza vaccination in model mice; they found that microneedle vaccination improved lung IgA titers, cellular immune responses and antibody-secreting cells. More importantly, virus clearance was more effective. The authors concluded that dissolvable microneedle patches provided not only practical advantages compared with conventional intramuscular injections but also better protective immunity [17]. McHugh et al. embedded microparticles containing near-infrared quantum dots in dissolvable microneedles. Rats with or without a vaccine were administrated with these microparticles; the vaccinated rats could be identified by exposing the rats to near-infrared light for quantum dot detection [96]. Microneedles are also used to deliver vaccines and antiretroviral drugs for the prevention and treatment of AIDS due to their convenience and could provide higher antibody levels [40,97]. Boopathy et al. fabricated microneedles encapsulating antigen ovalbumin (OVA) in regenerated SF protein with a dissolving PAA polymer backing. After the microneedle is applied to the skin, the entrapped vaccine is slowly released into the mice. Immunogenicity was further confirmed using an intact envelope HIV trimer immunogen. Additionally, the authors found that MN-based vaccination achieved 1300-fold higher antibody titer than equivalent intradermal injections (Figure 12A) [98]. In view of the current COVID-19 pandemic, Kim et al. treated COVID-19 mice with percutaneous injection of a recombinant SARS-CoV-2 S1 vaccine with dissolvable microneedles and tested the immunogenicity. They found that soluble microneedles containing a SARS-CoV-2 S1 subunit vaccine elicited a strong antigen-specific antibody response at 2 weeks after immunization. The immunogenicity of the microneedle array vaccine was maintained after gamma radiation sterilization [99]. Furthermore, vaccines can also be approached with nano carriers. For instance, Niu et al. applied hollow microneedles to deliver model antigen OVA and Toll-like receptor (TLR) agonists imiquimod and monophosphoryl Lipid A by entrapping the drugs inside the PLGA NPs. Compared to conventional subcutaneous injection, microneedle transdermal administration shows significantly higher NP concentrations in the draining lymph nodes with stronger immune responses. This also demonstrates that microneedles with nanoparticles for drug delivery have much better targeting and therapeutic effects in contrast to naked vaccines [100].

### 4.5. Biosensors

Microneedles are also used in sensors for two purposes: to serve as active ingredients of biosensors and to sample and deliver biological fluids to biosensors [103]. Compared to other continuous monitoring devices, microneedle biosensors have the following advantages: (1) MNs are less invasive to the skin because of their small size; (2) they have less biofouling effects for their replaceable capacity; (3) MNs provide larger electrode surface areas for larger currents; (4) MNs allow less costs but are an accurate device, as they can determine glucose concentrations in dermal ISF, which is similar to that of the blood. (5) The wound created by the microneedle sensor after removal can recover within 24 h [104,105]. Romanyuk et al. collected biomarkers with microneedles for analysis. All patches were weighed before and after being applied to the skin, which showed that the tissue fluid extracted from the microneedle patches was 0.84 ± 0.24 mg, and the absorption efficiency increased with collection time and number of cycles (Figure 12B) [101]. Sharma et al. assessed the continuous glucose monitoring microneedle devices in 14 healthy individuals and 10 patients with type I diabetes. The results showed that this device could provide clinically acceptable results lasting for 24 h with no inflammation and minimal discomfort. However, in this study, part of the microneedles showed no functionality post in vivo studies, and this may be due to the damage of gamma-ray irradiation for sterilization, which should be noted in further studies [105]. Using antibody/antigen binding to micro projection array (MPA), Kendall et al. developed a series of immunosensors for capturing skin protein biomarkers without extracting and collecting blood samples. A key step is to extract sufficient specific proteins from the skin to achieve high diagnostic sensitivity in a short period of time. According to Kendall et al., increasing needle column density and array size significantly increases biomarker capture while maintaining similar tolerance levels (Figure 12C) [102,106].

### 4.6. Other Potential Applications

#### 4.6.1. Pain Therapy

Lidocaine, a widely used local anesthetic, is usually used alone or in combination with other drugs to treat preoperative and postoperative procedural pain. Kathuria et al. reported a microneedle loaded with lidocaine with fast and sustained release of lidocaine, which helps relieve the acute and chronic pain of injection [73]. Furthermore, Xie et al. applied calcitonin-gene-related peptide receptor antagonist (anti-CGRP) peptides entrapped in dissolvable microneedles to alleviate chronic nerve pain caused by inflammation after trauma. Then, these microneedles were applied to rats with neuropathic pain caused by nerve injury, diabetic neuropathy and neurogenic inflammatory. Results of thermal and mechanical tests proved that MN loaded with anti-CGRP peptide could produce safe and effective analgesic effects [107].

#### 4.6.2. Mucosa Therapy

Caffarel-Salvador et al. reported the delivery of human insulin and hGH to the buccal mucosa by dissolvable MNs. Due to the use of MNs, quite less pain was claimed through a visual analog pain scale score collected from 100 volunteers [5]. Additionally, as reported in another work by Ma et al., doxorubicin (DOX) was firstly loaded to PLGA nanoparticles, and the nanoparticles were coated on microneedles to be applied to the cancerous region in oral phantom tissue. These coated microneedles had the advantage of not leaking the DOX and causing fewer side effects over traditional injections [108].

#### 4.6.3. Eye Therapy

Drug administration in the eyes requires multiple doses due to biofilm limitations. Currently, many studies have been reported on the application of microneedle to deliver drugs to the eyes due to the minimal invasive characteristics and higher ocular bioavailability of the microneedle [109]. Patel et al. delivered particles with diameters of 500 nm to 1000 nm loading with sulforhodamine B into the suprachoroidal part (pig, rabbit and human cadaver eyes) by hollow microneedles. Based on the particle diffusion radius, they showed that the microneedles helped the particles spread at the back of the eye. They also discussed the effect of microneedle length and particle size on the success rate of injection in the suprachoroidal space. Additionally, the longest microneedles (1000 μm) had no significant dependence on particle size, while shorter microneedles limited the diameters of the particles [9]. Roy et al. designed and manufactured a PVA-PVP patch that combined contact lenses and microneedles. The patch was formed by micromolding and loaded with pilocarpine hydrochloric acid. They compared the penetration of the PVA-PVP patch with the pilocarpine solution in the removed human corneas. It was found that pilocarpine had a better releasing ability in microneedles with better retention in the cornea compared to that in solution form [110].

## 5. Pitfalls

### 5.1. Mechanical Strength and Reproducibility

**Figure 13:**
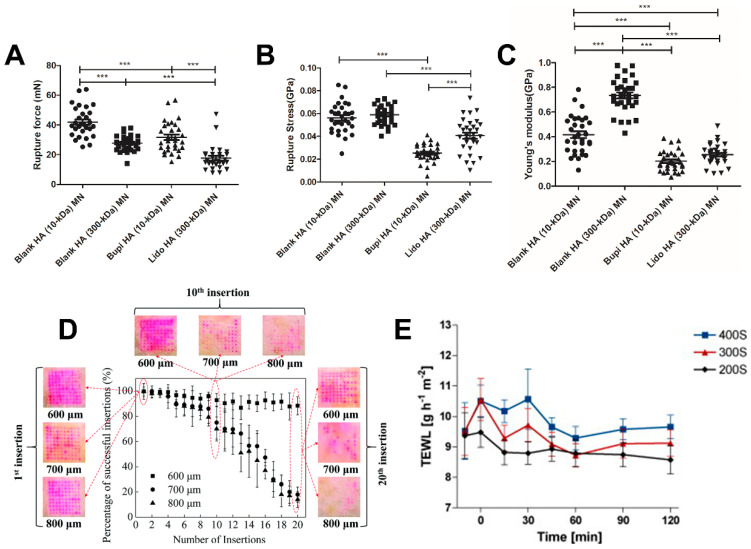
(**A**) Rupture forces of four types of MNs. (**B**) Rupture stress of four types of MNs. (**C**) Young’s modulus of four types of MNs. (**D**) The relationship between PLA MN insertion success rate and insertion times with different heights (600, 700 and 800 μm). (**E**) Skin irritation and barrier disruption were assessed by TEWL (200S as 200 µm, 300S as 300 µm, 400S as 400 µm). In (**A**–**C**), one-way ANOVA analysis is applied, and the significant difference is indicated by * *p* < 0.05, ** *p* < 0.01, *** *p* < 0.001. (Image was reproduced with permission from [111,112,113]).

Microneedles require certain levels of mechanical strength and toughness to ensure penetration of the stratum corneum without deformation and fracture. The size of microneedles should also be carefully designed to minimize needle dimensions while maximizing drug load. Moreover, different application areas and microneedle sizes require different manufacturing processes. According to Du et al., their study suggests that the molecular weight of the polymer and the loading types of the drug had significantly similar effects on the mechanical properties of microneedles. They applied HA with different molecular weights (10 kDa, 300 kDa) to fabricate the microneedles, and two HA MNs (10 kDa, 300 kDa) loaded with bupivacaine and lidocaine, respectively. The rupture forces (Figure 13A), stress (Figure 13B) and Young’s modulus (Figure 13C) of the four microneedles were measured. As a result, HA (10 kDa) MN and HA (300 kDa) MN had similar mechanical strength, better than their drug-loaded microneedles, respectively. In addition, other types of polymers affect the mechanical strength of dissolvable MN [111]. Wang et al. implied the property of mechanical and ability of MNs with PVA, hyaluronic acid (HA), chitosan and gelatin as the substrate, respectively, under different humidity conditions. Under the same manufacturing method (micromolding), the four microneedles have the same size and height. However, compared to the other three MNs, PVA-MN shows more sensitivity to humidity conditions. Additionally, the mechanical strength of PVA MNs could only keep within 10 min when the humidity condition was about 40%, whereas the other three could maintain their structure even after 120 min [42]. Thus, it is also necessary to select the appropriate polymer as the substrate of the microneedle. 

However, the designed structure of MNs (e.g., tip length) is also very important for the usage and safety of MNs. Li et al. fabricated polylactic acid (PLA) microneedles with different heights (600, 700 and 800 μm) to insert them in porcine cadaver skin multiple times. The redness of the skin was taken as evidence of successful insertion (Figure 13D). Especially for the MN with a height of 600 μm, the number of insertions had little effect on the success injection rate. However, for MNs with heights of 700 and 800 μm, by the twentieth insertion into the skin, the successful insertion percentage decreased to about 20%. Additionally, some MN tips are very easily damaged due to the long body of the MNs [113]. Chen et al. compared the efficiency of insulin delivery between metal microneedles (124, 248 μm) and polymer microneedles (248, 337 and 445 μm) of different lengths by optimal scaling analysis. They found that insulin concentrations in mice treated with microneedles were significantly higher than in untreated mice. Besides, the results showed that the longer the microneedle length, the higher the insulin concentration in mice [114]. Similarly, Chandra et al. observed the relationship between the amount of drug delivered and the thickness and surface area of the skin, which indicated the role of the geometric parameters of the microneedles [115].

Furthermore, the vibration-minimized micromachined microdevice was applied to manufacture PDMS molds. After using the PDSM mold many times, the actual measured value of the microneedle was still very close to the theoretical measured value of the mold. However, the fabrication precision about the height, base diameter and precision of the interbase spacing of the PLGA MNs were investigated to have ±0.18%, ±0.45% and ±0.22% deviation, respectively [116]. The fracture forces of the polymer microneedles in both directions (axial and transverse) were measured using a micromechanical test machine, for they could predict the bending behavior during the insertion into the skin [116]. In order to have a better command of the insertion of microneedles into the skin, Shu et al. designed a model that reflects the skin condition in vivo. They also found that increasing skin tension could help improve the convenience and effectiveness of microneedle insertion, which was argued by previous studies [117].

### 5.2. Safety

Drug delivery by microneedles is achieved by increasing skin permeability through micropores. If the micropores are not closed after administration, microorganisms or viruses may enter the body and cause infections. Instead, studies showed the risk of the impact of microneedles is low. Several investigations on whether microneedles could cause skin safety issues or not have been explored. Vicente-Perez et al. inserted two microneedles into hairless mice every week for 5 weeks. No significant changes in skin appearance or skin barrier function were observed in mice regardless of microneedle formulation, needle density or the number of applications. At the end of the study, there were no significant differences in serum biomarkers of stimulation/inflammation, infection and immunity between the experimental and control groups. Zvezdin et al. applied hyaluronic acid dissolvable microneedle patches (length, 450 µm; average penetration depth, 26 µm) into the epidermis of excised skin explants. These microneedles noninvasively penetrated the dermis layer. After the skin puncture with microneedles, 17 sites of microneedle puncture were observed immediately, while fours sites were found 2 h later. The differences in the depth of epidermal penetration did not affect cell viability [118]. In subsequent clinical trials, the micro-needle patches were applied to the periorbital area for 25 min twice per week for 3 weeks. Zvezdin et al. found that some subjects developed mild to moderate congestion and edema immediately after the treatment, which were caused by mechanical microdamage to the skin by the penetration of microneedle structures into the epidermis. In addition, the mean time for congestion to stabilize was 31.38 ± 2.46 min. Side effects such as post-inflammatory hyper-pigmentation, epidermal burns and scar formation were not observed [119]. Bal et al. used 200, 300 and 400 µm microneedles on the upper arms of 18 volunteers to investigate the effect of microneedle length on transdermal water loss (TEWL) for assessing skin irritation and barrier disruption. In general, a high TEWL value is associated with skin damage, while a low TEWL value indicates less damaged or intact skin [120]. The TEWL and redness values after treatment with 400 µm microneedle were significantly higher than those after 200 µm microneedle treatment. Longer microarrays increased TEWL values and irritation of the skin. However, compared to the control treatment, 300 µm did not significantly increase TEWL (Figure 13E) [113]. In the study by Y. Han et al., the TEWL was significantly higher after 250 µm microneedles than after 150 µm microneedles; it took 24 h to recover to the baseline value in the 150 µm group and 48 h in the 250 µm group [121].

In addition, microneedles need to be sterile to avoid side effects, and this would limit the fabrication process of microneedles. For example, solid metal microneedles can be sterilized by heat, while dissolvable microneedles require gamma-ray sterilization [96,122]. In addition, McCrudden et al. investigated the effects of microneedles loaded with drugs by three sterilization methods (steam sterilization, dry heat sterilization and ionizing radiation (gamma) sterilization). After assessing the mechanical press’s effects on the microneedles, they found that the first two methods destroyed the formulations of the drug-loaded MNs, while gamma radiation sterilization damaged the protein stability and influenced the release of ibuprofen sodium [123]. Thus, it is necessary to consider whether microneedles can retain their original properties after sterilization or if it is better to choose antibacterial materials to form MN.

## 6. Conclusions and Future Outlook

For more than 40 years following the invention of microneedles, various methods and materials have been used to fabricate microneedles for better effects in transdermal drug delivery. Materials used in microneedle fabrication have evolved from metals to silicon and then polymers. Microneedle fabrication methods have been optimized and improved to be more flexible and efficient, evolving from lithography to micromolds and 3D printing [124]. Although 3D printing can produce personalized microneedles with high drug-loading capacity, this method is not yet widely used in clinical practice due to the high cost and complex production process. It has also been found that different types of microneedles are suitable for different drugs. With further investigation, it has been found that microneedles can also be used to detect biomarkers, such as blood glucose. Taken together, we hope that microneedles can be further developed to build a solid bridge between drugs and the human body. Microneedle patches that are effective in animal models need to be studied in more clinical trials to confirm their effectiveness in humans.

## Figures and Tables

**Table 1:** Microneedles for commonly used vaccines [95].

Drug-Coated Microneedles	Dissolvable Microneedles	Hollow Microneedles
Adenovirus	Adenovirus	Anthrax
Bacillus Calmette–Guérin	Amyloid beta peptide	*Clostridium botulinum*
Chikungunya virus	Diphtheria	Influenza
Hepatitis B	HIV	Japanese encephalitis
Hepatitis C	Influenza	Poliovirus
Herpes simplex virus	Malaria	Rabies virus
Human papilloma virus	Measles	*Staphylococcus aureus*
Influenza	Poliovirus	*Yersinia pestis*
Malaria		
Modified Vaccinia virus Ankara		
Rotavirus		
